# Supplementary material for: Layer-by-layer self-assembly of pillared two-dimensional multilayers
Source: Nat Commun. 2019 Jun 11;10:2558. doi: 10.1038/s41467-019-10631-0 (PMC6560128; doi:10.1038/s41467-019-10631-0)
Supplement: Supplementary file 1 — Supplementary Information [file 41467_2019_10631_MOESM1_ESM.pdf]

Supplementary Information for

Layer-by-Layer Self-assembly of Pillared Two-dimensional Multilayers

Weiqian Tian[1, 2†], Armin VahidMohammadi[3†], Zhen Wang[1, 2], Liangqi Ouyang[1], Majid Beidaghi[3, *], Mahiar M. Hamedi[1, 2*]

[1] Department of Fibre and Polymer Technology, KTH Royal Institute of Technology, Teknikringen 56, 10044 Stockholm, Sweden

[2] Wallenberg Wood Science Centre, Department of Fibre and Polymer Technology, KTH Royal Institute of Technology, Teknikringen 56, 10044 Stockholm, Sweden

[3] Department of Mechanical and Materials Engineering, Auburn University, Auburn, Alabama 36849, United States.

(†) These authors contributed equally to this work.

(*) Corresponding authors: Mahiar M. Hamedi (mahiar@kth.se); Majid Beidaghi (mbeidaghi@auburn.edu)

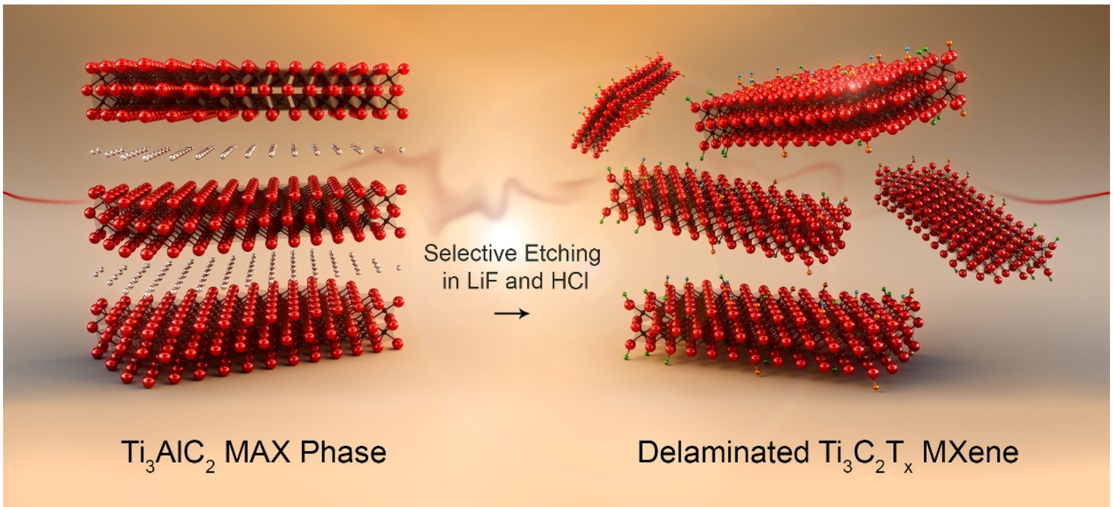

Supplementary Figure 1 | Schematic illustration of $Ti_3C_2T_x$ MXene synthesis. Selective etching of Al atoms from Ti_3AlC_2 MAX phase in a LiF and HCl mixture.

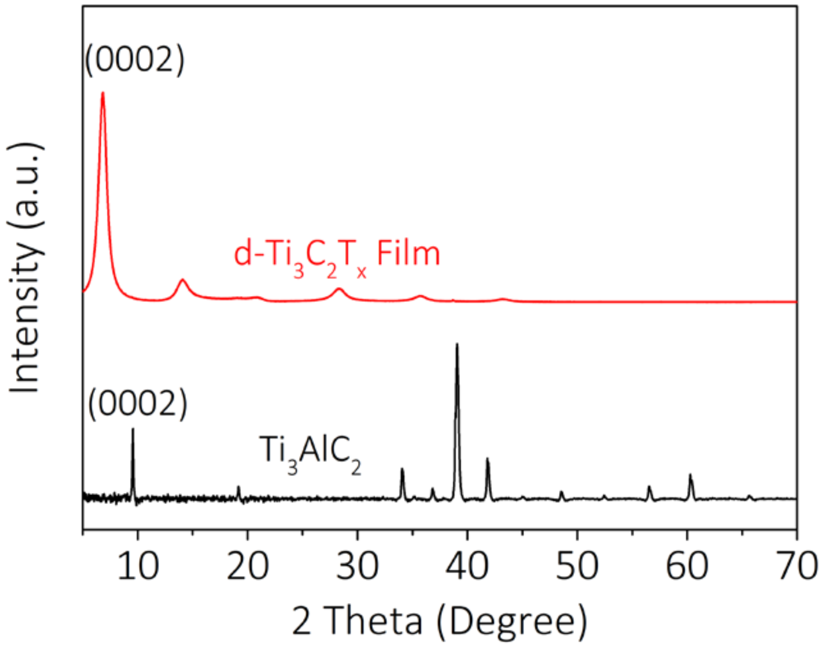

Supplementary Figure 2 | XRD characterization of MAX phase and MXene. XRD Spectra of the Ti_3AlC_2 and delaminated (d-$Ti_3C_2T_x$) paper produced by vacuum filtration of MXene solution. After exfoliation of MAX phase and successful delamination of etched MXene particles, the (0002) peak of the Ti_3AlC_2 at around 9.57° broadens and shifts down to 6.85° for the MXene. All the remaining characteristic peaks of MAX phase are removed after etching and exfoliation in the acidic etchant.

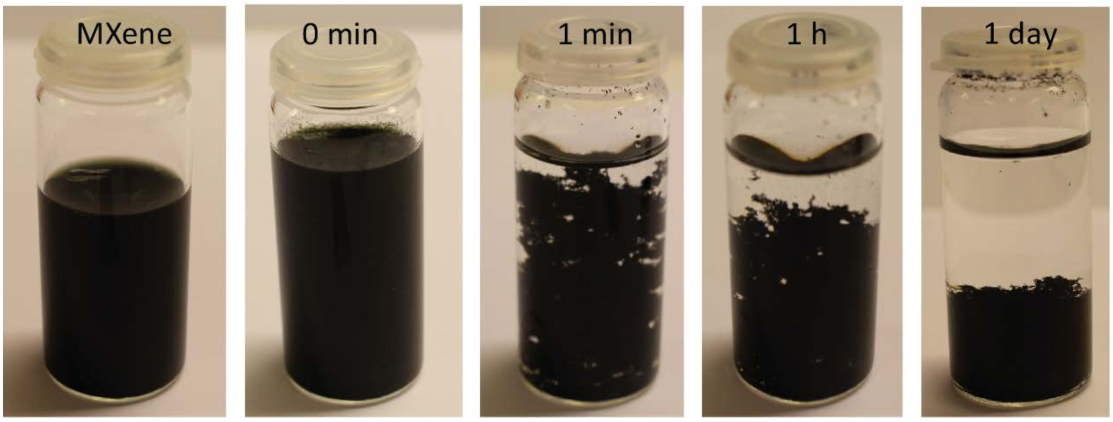

Supplementary Figure 3 | Self-assembly formation of MXene/TAEA hybrids after mixing the MXene dispersion with TAEA solution. The photographs are for 1 g/L MXene dispersion, and the MXene/TAEA hybrids at the different time after mixing, from left to right.

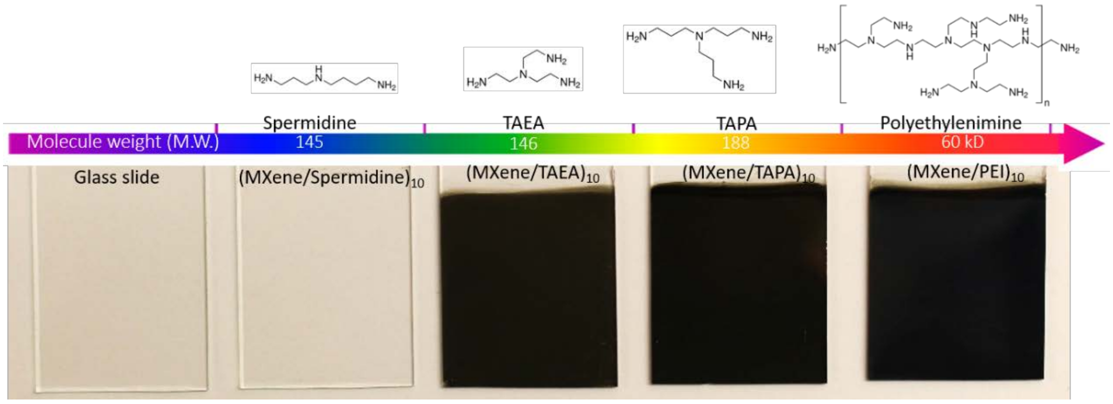

Supplementary Figure 4 | LbL self-assembly of MXene flakes using different cationic counter phases. This figure clearly shows that diamino small-molecule such as spermidine does not work for this LbL process. While the triamino molecules of TAEA and tris(3-aminopropyl)amine (TAPA) both work well. We therefore, inferred that the triamino molecule is the minimum requirement that can lead to LbL self-assembly, and the TAEA is the smallest triamino molecule that can be chosen for LbL self-assembly process.

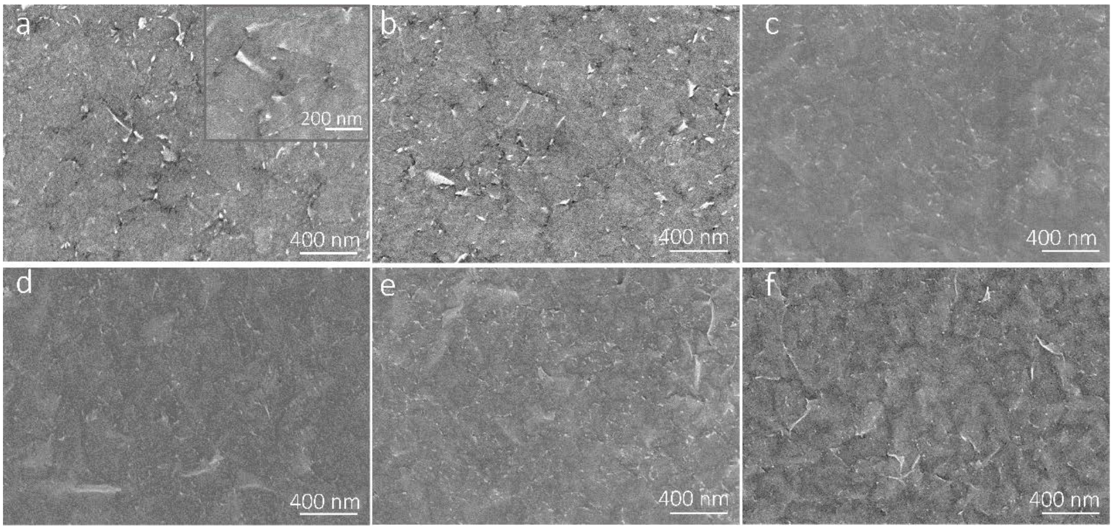

Supplementary Figure 5 | Top-view SEM images of $(MXene/TAEA)_n$ multilayers with the different bilayer numbers: (a) $(MXene/TAEA)_1$, (b) $(MXene/TAEA)_3$, (c) $(MXene/TAEA)_6$, (d) $(MXene/TAEA)_{10}$, (e) $(MXene/TAEA)_{20}$, and (f) $(MXene/TAEA)_{30}$. Inset in (a) shows a higher magnification image of $(MXene/TAEA)_1$. These top-view SEM images showed face-to-face stacking between individual nanoflakes in the range from n=1 to n=30 bilayers, and the formation of slit-shaped pores that can act as fast ion diffusion channels.

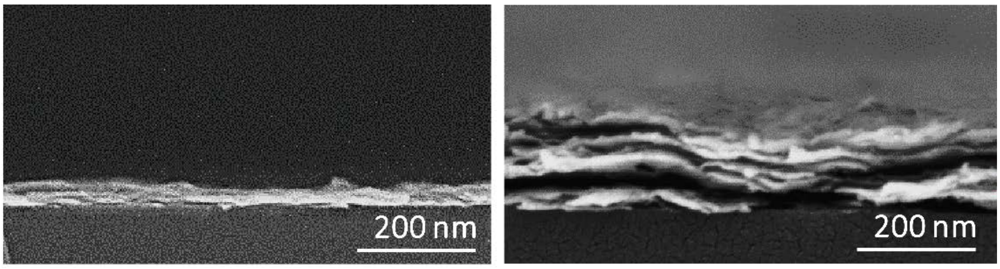

Supplementary Figure 6 | Cross-sectional SEM images of multilayers on silicon wafers. (a) $(MXene/TAEA)_6$ and (b) $(MXene/TAEA)_{30}$.

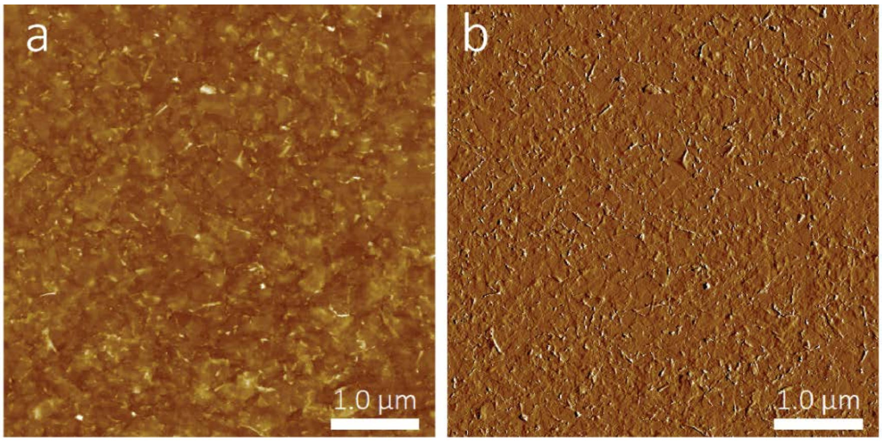

Supplementary Figure 7 | AFM image of (MXene/TAEA)$_6$ on a silicon wafer. (a) Height image and (b) corresponding peak force error image, with a roughness R_a of 2.45 nm in an area of 2 µm × 2 µm.

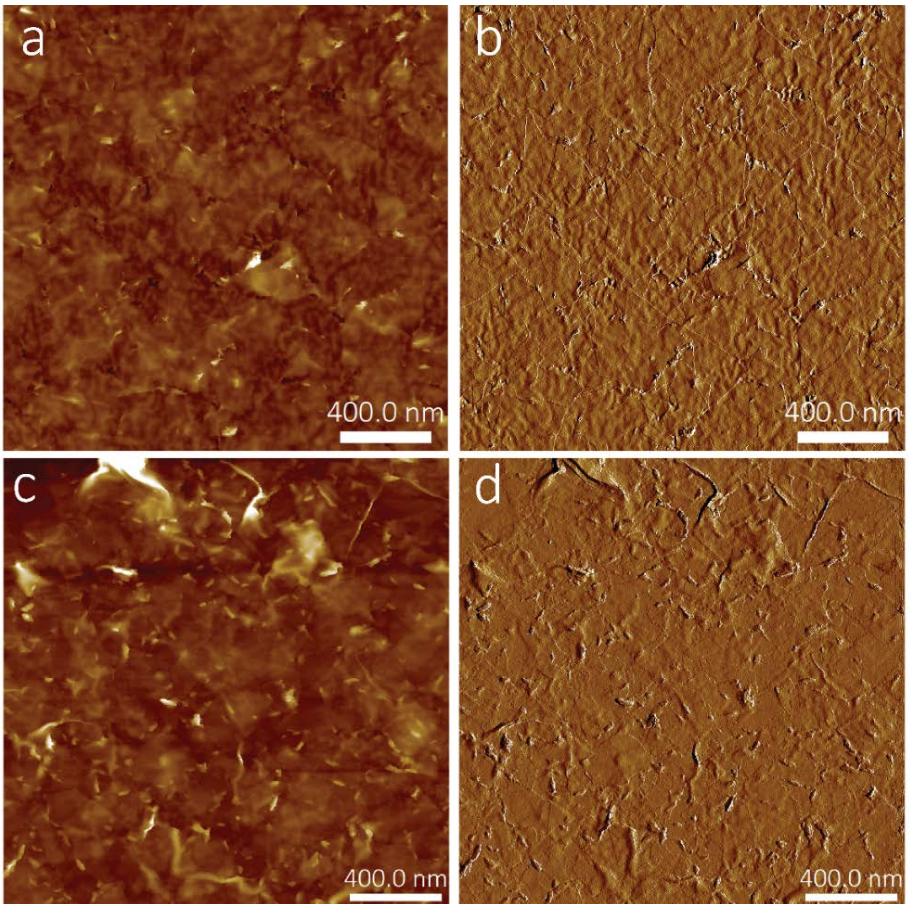

Supplementary Figure 8 | AFM height and corresponding peak force error images. (a, b) (MXene/TAEA)$_1$ onto a PET film with a roughness R_a of 1.91 nm in area 2 µm × 2 µm, and (c, d) (MXene/TAEA)$_{30}$ onto a silicon wafer with a roughness R_a of 3.66 nm in an area of 2 µm × 2 µm.

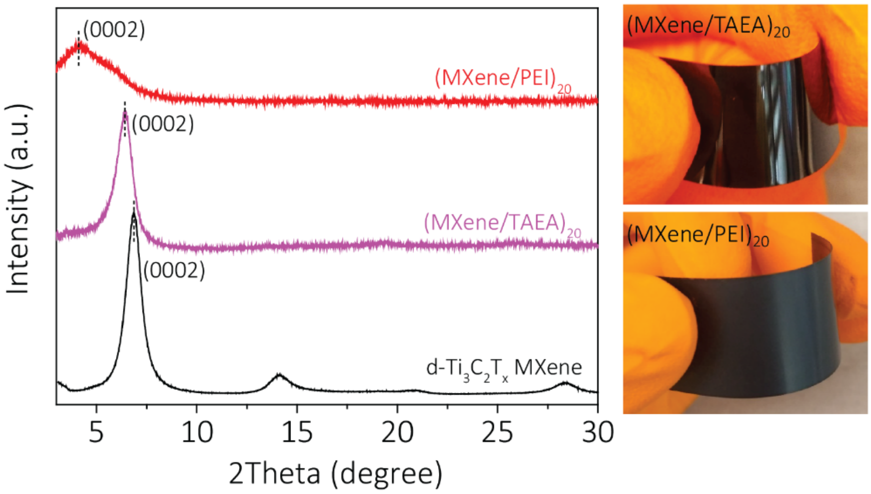

Supplementary Figure 9 | XRD patterns of $(MXene/PEI)_n$ compared with MXene film and $(MXene/TAEA)_n$, and digital photographs of the $(MXene/TAEA)_{20}$ and $(MXene/PEI)_{20}$. The position of (0002) peak of $(MXene/PEI)_{20}$ shifts from 6.89° of prisitne MXene films to 4.10°, corresponding to an increase of 8.71 Å in the interlayer spacing from 12.81 Å up to 21.53 Å.

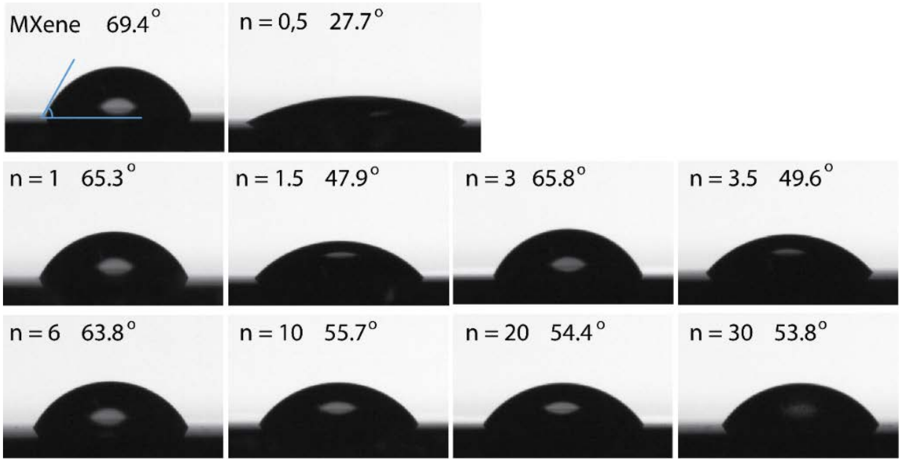

Supplementary Figure 10 | Contact angles of MXene and $(MXene/TAEA)_n$ with different bilayer numbers. “n=1" means that the MXene is the last layer of the multilayers, while “n=0.5” indicates that TAEA is the last layer. The photographs were taken after 20 s resting the water droplet on the surfaces.

It is noteworthy that the hydrophilicity of MXene multilayers increased with the multilayer number n, which was ascribed to the increase of surface roughness. While the multilayers finished with the TAEA as the last layer showed smaller contact angles than those of the multilayers finished with the MXene, suggesting a higher wettability for TAEA layer.

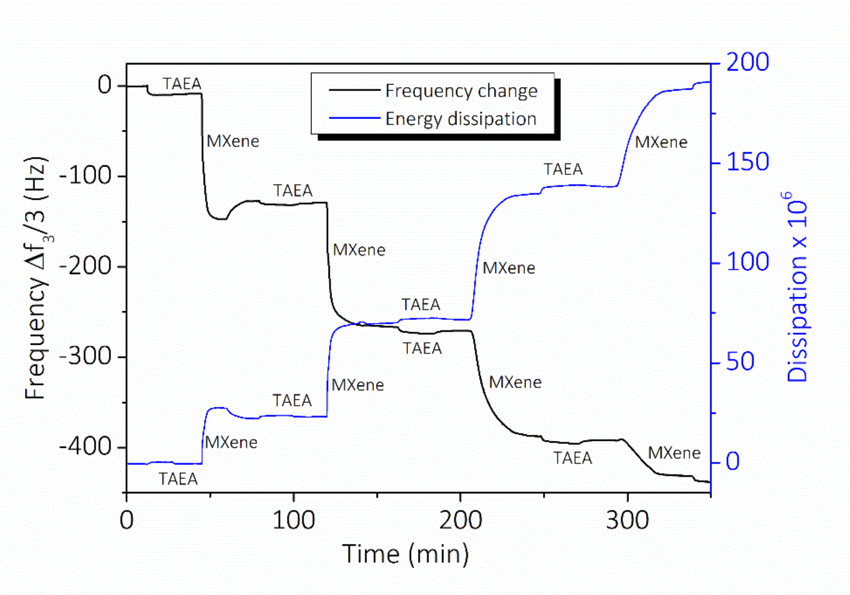

Supplementary Figure 11 | Quartz crystal microbalance (QCM) measurement of the (MXene/TAEA)$_n$ multilayers. QCM data further showed the successful formation of the (MXene/TAEA)$_n$ multilayers on a silica model surface.

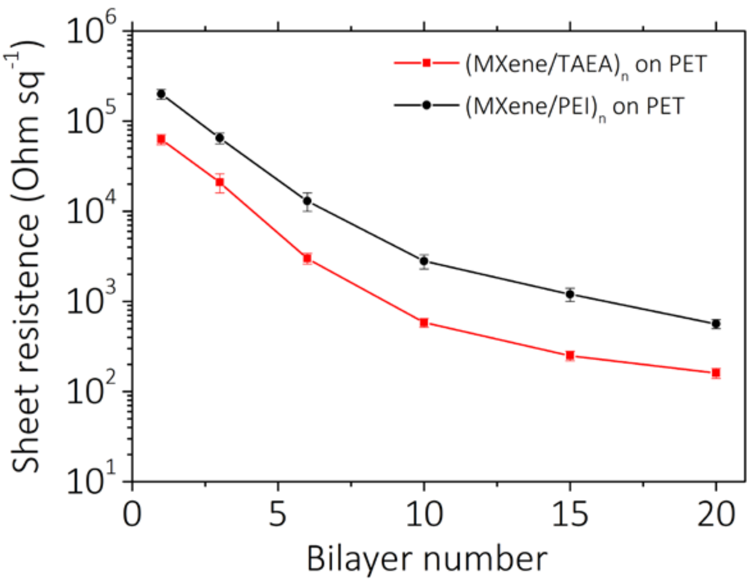

Supplementary Figure 12 | Sheet resistances of (MXene/TAEA)$_n$ and (MXene/PEI)$_n$ on PET films.

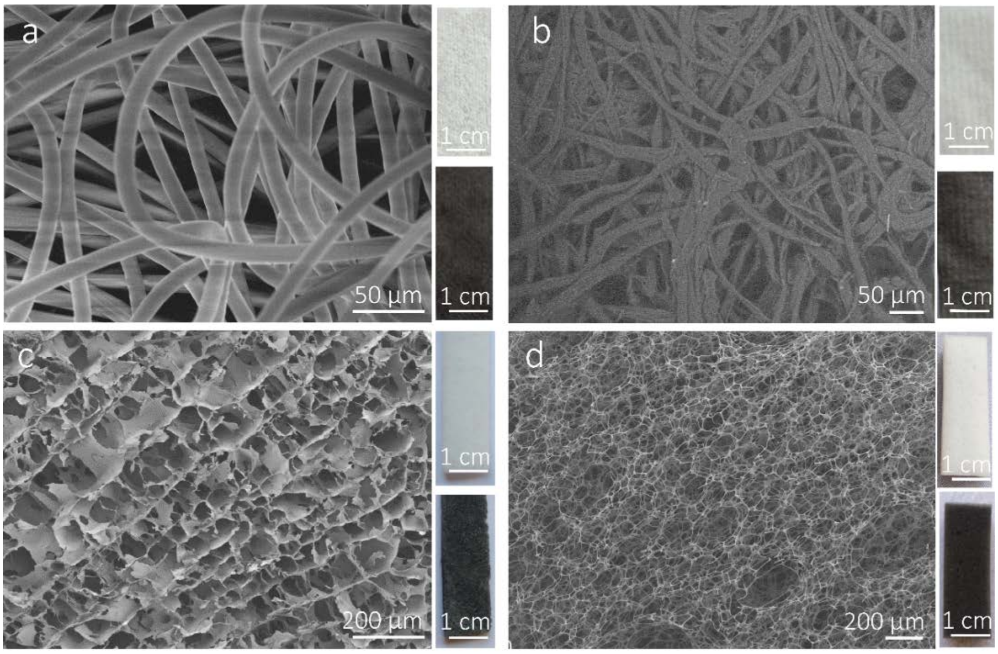

Supplementary Figure 13 | SEM images of the porous substrates and digital photographs of the substrates assembled by MXene before (up) and after (down). (a) nonwoven, (b) cellulose paper, (c) CNF aerogel, and (d) melamine foams.

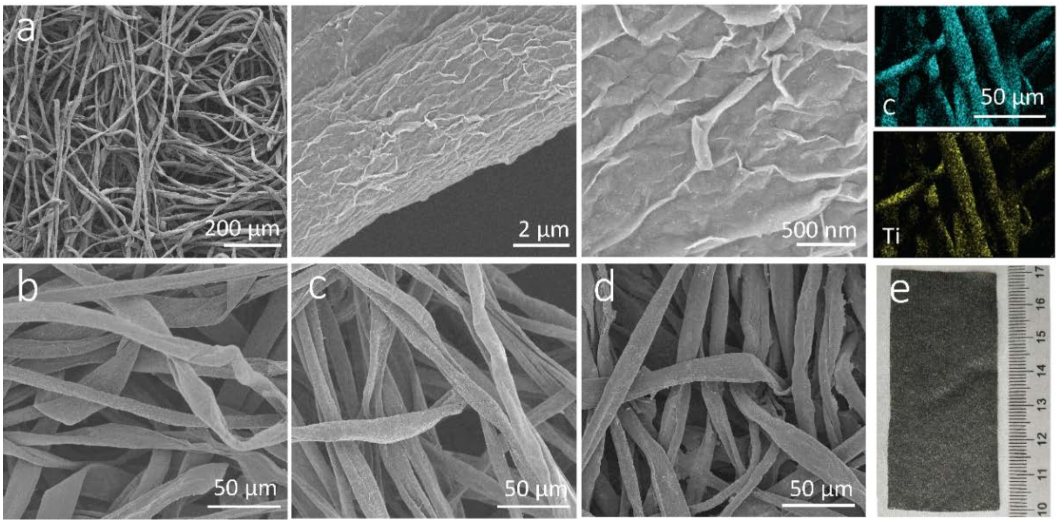

Supplementary Figure 14 | SEM images and EDS elemental mappings of the (MXene/TAEA)$_n$ assembled onto a cotton fiber-based nonwoven. (a) (MXene/TAEA)$_6$, (b) (MXene/TAEA)$_1$, (c) (MXene/TAEA)$_{10}$, and (d) (MXene/TAEA)$_{20}$ multilayers. (e) Digital photograph of a larger-scale (MXene/TAEA)$_6$ on nonwoven. The (MXene/TAEA)$_n$ on nonwoven still showed the conformal growth of (MXene/TAEA)$_n$ multilayers by increasing the bilayer number.

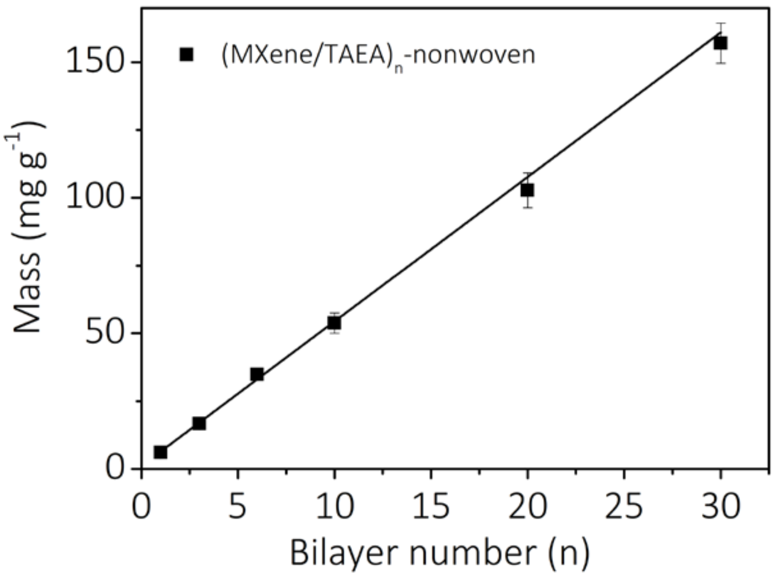

Supplementary Figure 15 | The mass loading of (MXene/TAEA)$_n$ multilayers on the nonwoven based on the mass of nonwoven.

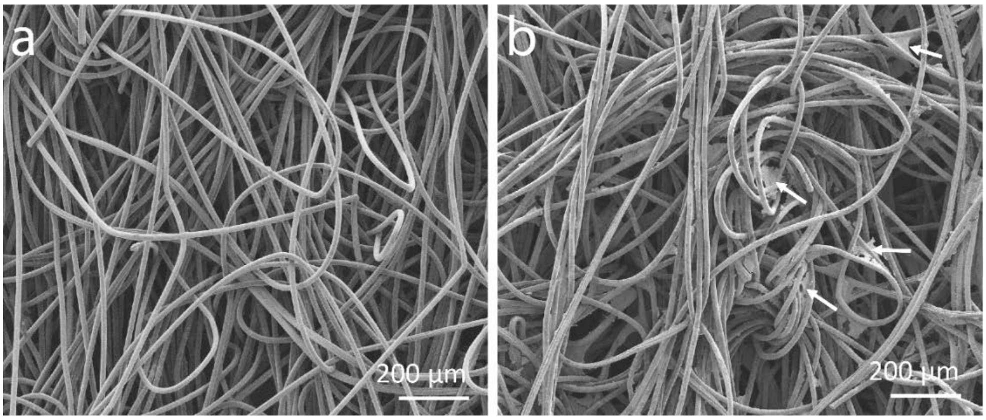

Supplementary Figure 16 | SEM images of (MXene/TAEA)$_{20}$ on nonwoven. The samples were assembled with vacuum-assisted step (a) and without the vacuum-assisted step (b) which shows that in some areas (indicated by arrows) the MXene flakes connect the gaps between adjacent fibers because of the lack of enough force to compel the redundant MXene flakes through the entire fiber nets. Vacuum-assisted spraying further minimized the assembly time and effectively removed the stagnant drips caused by the flow separation of the spray streamline from the leading-edge region of the fibers or the walls of the foams.

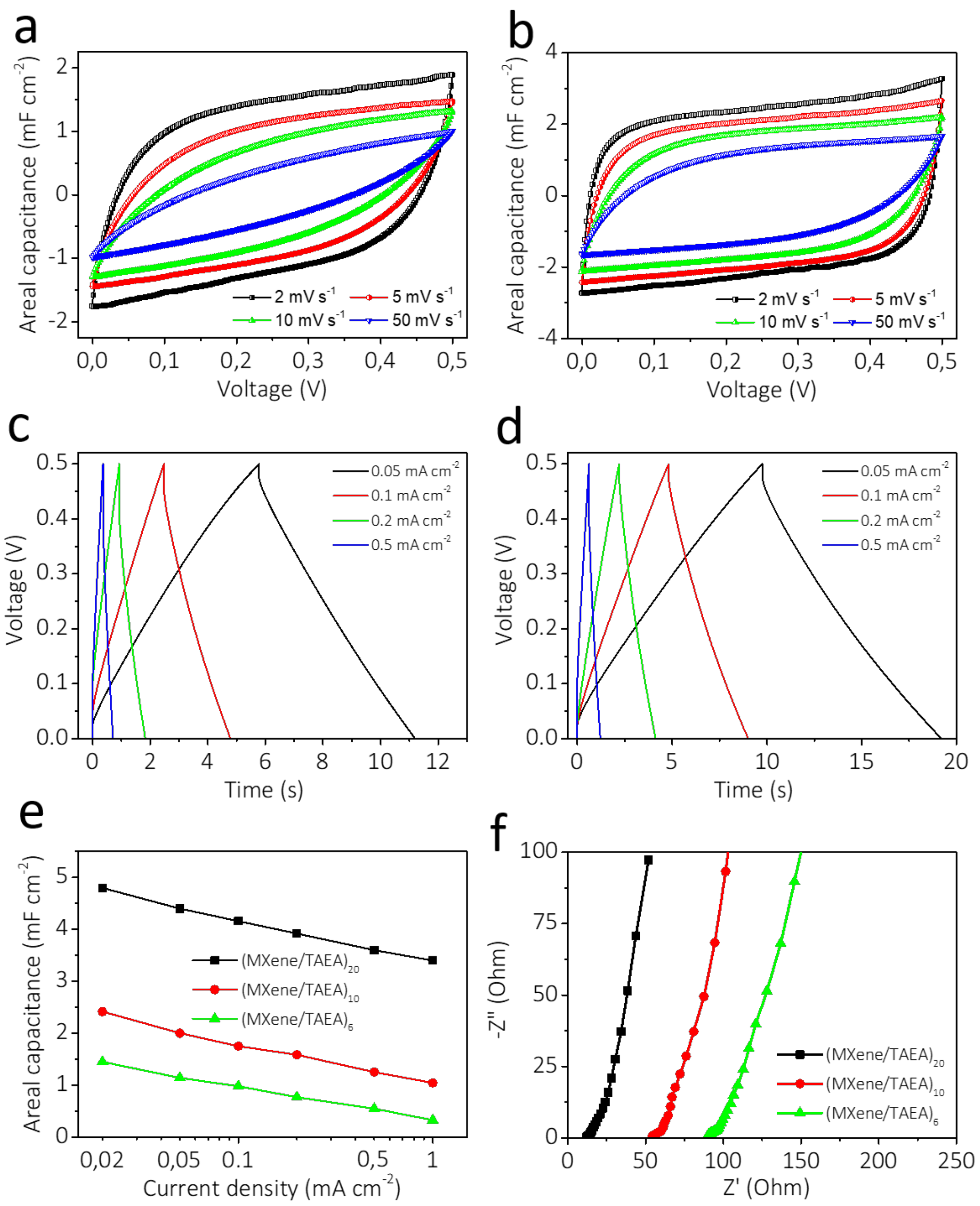

Supplementary Figure 17 | Electrochemical performances. CV curves at different scan rates and charge-discharge profiles at different current densities of (a, c) $(MXene/TAEA)_6$ and (b, d) $(MXene/TAEA)_{10}$-based solid-state supercapacitors. (e) The areal capacitance calculated from discharge curves at different current densities, and (f) Nyquist plots of $(MXene/TAEA)_n$-based solid-state supercapacitors. Our devices show small charge transfer resistances which are due to the unique LbL microstructures of $(MXene/TAEA)_n$. While the high equal series resistances (ESR) are mainly due to the fact that the $(MXene/TAEA)_n$ multilayers were used as both the active materials and the current collectors in the devices.

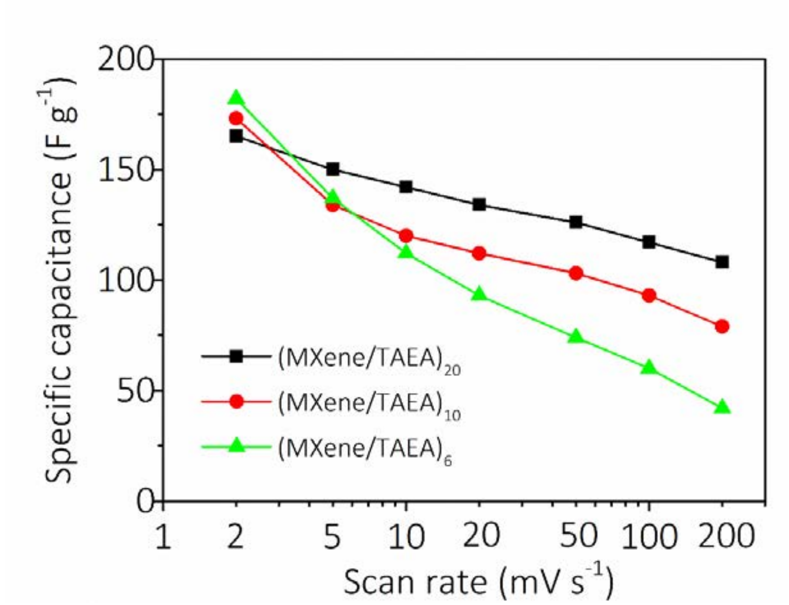

Supplementary Figure 18 | Gravimetric capacitances of (MXene/TAEA)$_n$-based solid-state supercapacitors at different scan rates.

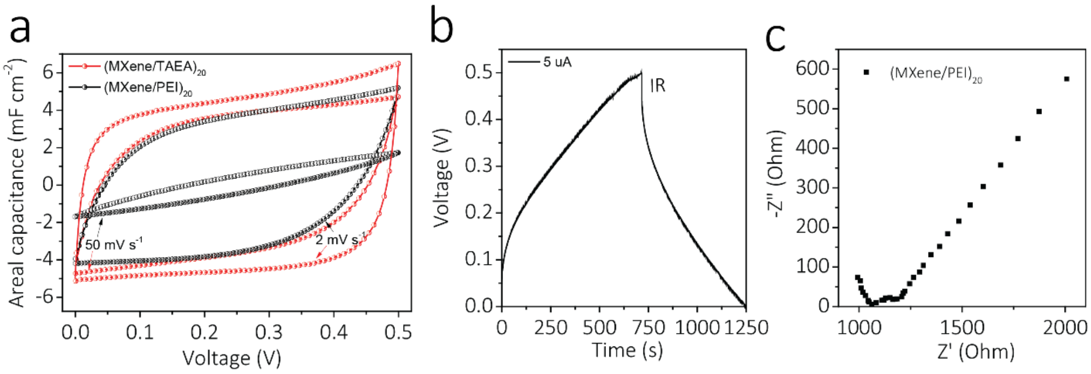

Supplementary Figure 19 | Electrochemical performances of (MXene/PEI)$_{20}$-based solid-state supercapacitors. (a) CV curves of (MXene/PEI)$_{20}$ at different scan rates compared with those of (MXene/TAEA)$_{20}$. (b) Charge-discharge profile of (MXene/PEI)$_{20}$ with a large IR drop. (c) Nyquist plots of (MXene/PEI)$_{20}$-based solid-state supercapacitors.

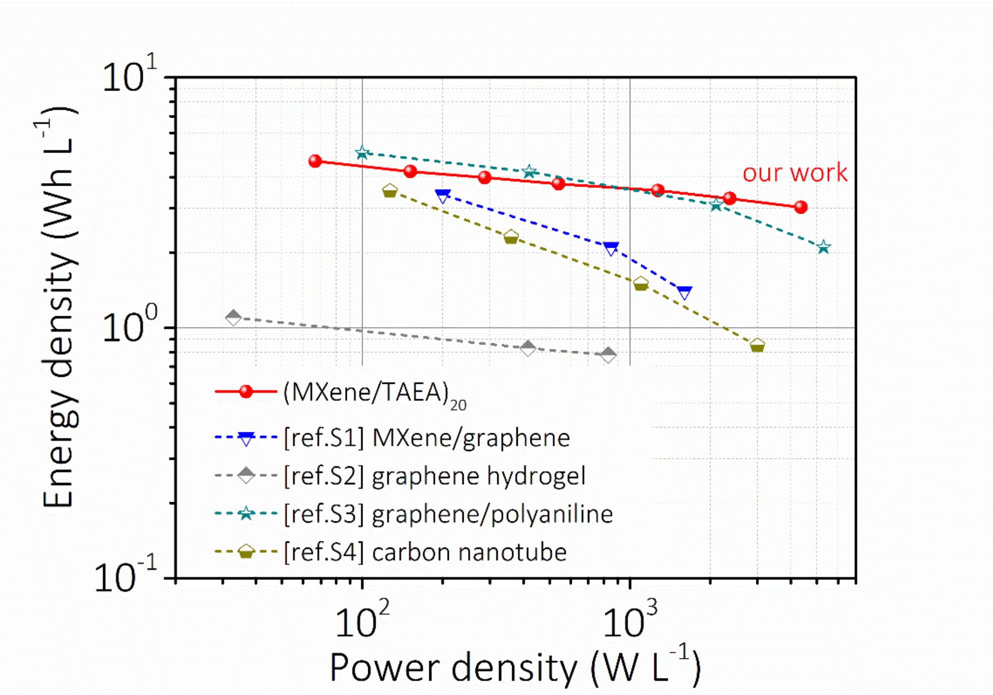

Supplementary Figure 20 | Ragone plot. The energy-power densities of $(MXene/TAEA)_{20}$-based solid-state supercapacitors compared to other reported solid-state supercapacitors with carbonaceous electrodes, based on total active electrode volume.

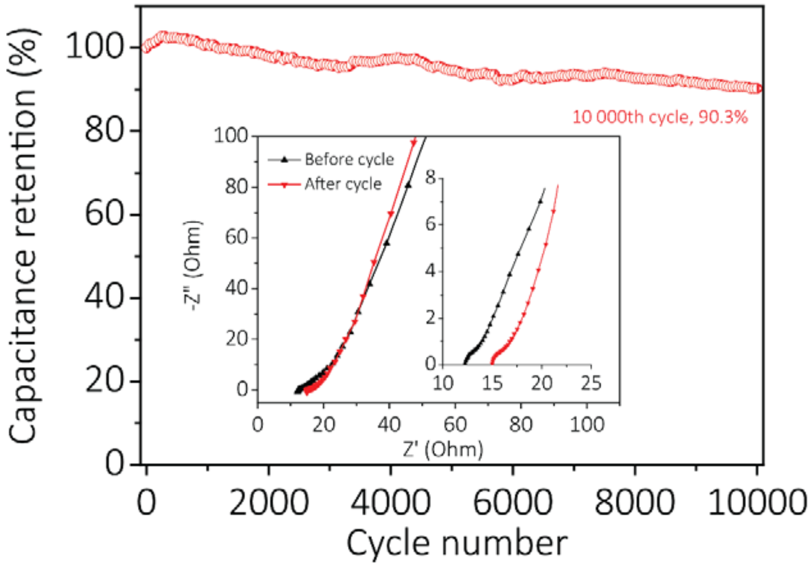

Supplementary Figure 21 | Cycle stability $(MXene/TAEA)_{20}$-based solid-state supercapacitors. Inset shows the Nyquist plots of $(MXene/TAEA)_{20}$-based solid-state supercapacitors before and after 10 000 cycles. After long cycling our device showed a small increase in equal series resistances.

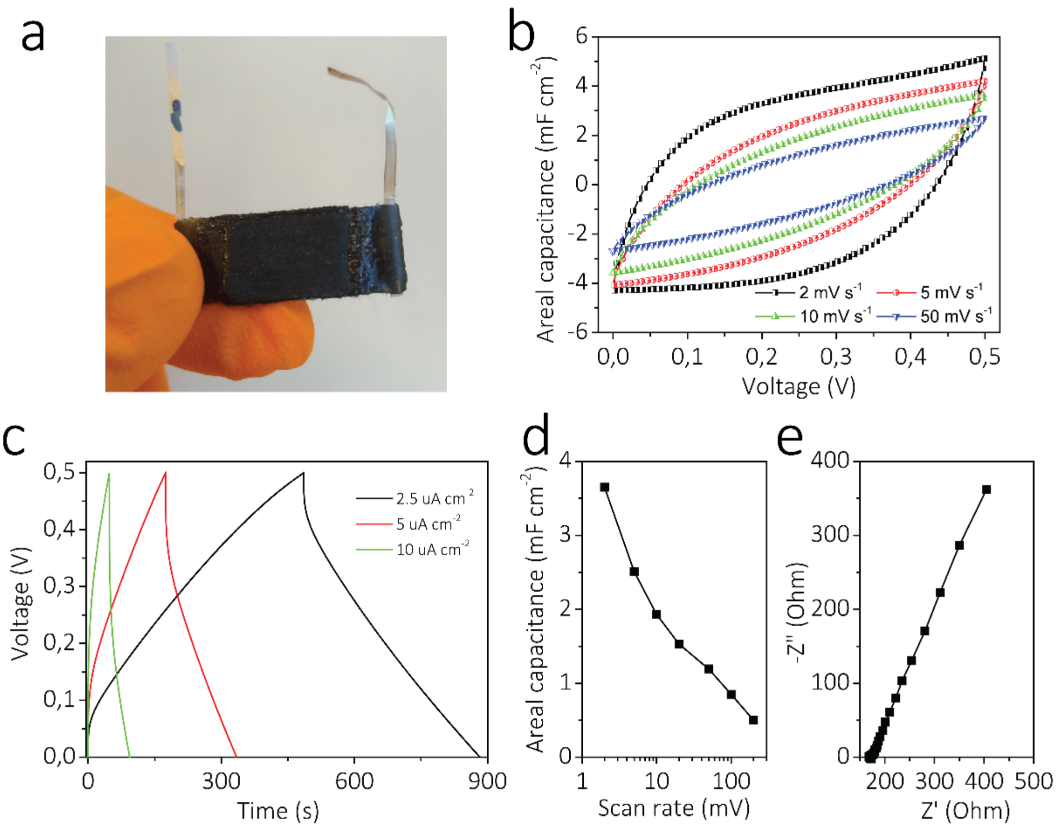

Supplementary Figure 22 | Electrochemical properties of the solid-state supercapacitors based on the (MXene/TAEA)$_{20}$ on nonwoven electrodes. (a) Digital photograph of the solid-state supercapacitors based on the (MXene/TAEA)$_{20}$ on nonwoven, (b) CV curves at different scan rates, (c) charge-discharge profiles at different current densities, (d) specific area capacitance at different scan rates, and (e) Nyquist plots of the solid-state supercapacitors based on the (MXene/TAEA)$_{20}$ on nonwoven.

The Quasi-rectangular CV curves and linear charge-discharge profiles show a capacitive behavior. The device shows a lower areal capacitance of 3.7 mF cm^{-2} at a scan rate of 2 mV s^{-1}, and a lower rate capability compared with that of (MXene/TAEA)$_{20}$ on PET. This is because the solid electrolyte PVA/H_2SO_4 cannot efficiently access all MXene multilayers on the complex porous matrix during the electrochemical proces, leading to the larger ESR and diffusion resistance of the device.

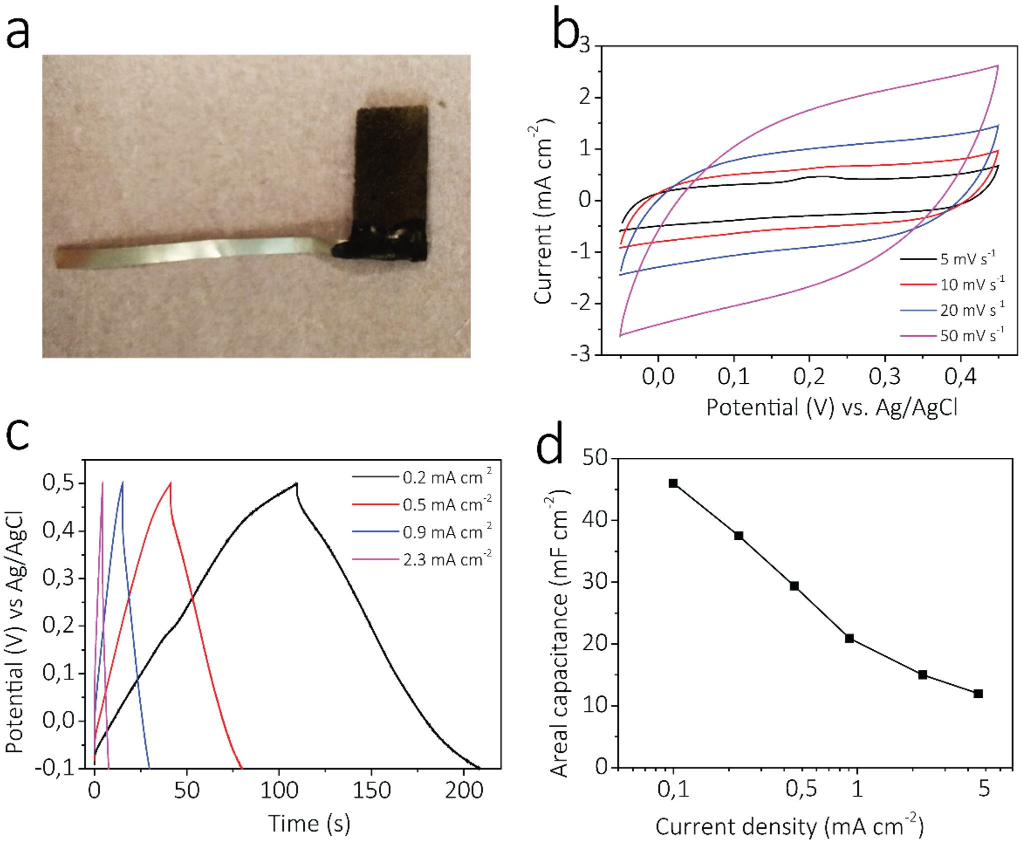

Supplementary Figure 23 | Electrochemical properties of the (MXene/TAEA)$_6$ on CNF aerogel. (a) Digital photograph, (b) CV curves at different scan rates, (c) charge-discharge profiles at different current densities, and (d) specific areal capacitance of (MXene/TAEA)$_6$ on CNF aerogel-based electrode in the three-electrode configuration using 1 M H_2SO_4 electrolyte.

We used a three-electrode configuration to test the electrochemical properties of (MXene/TAEA)$_n$ on CNF aerogel in 1 M H_2SO_4 electrolyte (Figure S16). Quasi-rectangular CV curves and linear charge-discharge profiles are characteristics of a capacitive behavior. The electrode displays a high areal capacitance of 46 mF cm^{-2} at a current density of 0.1 mA cm^{-2}. A capacitance of 12 mF cm^{-2} was still retained as current density was increased 45 times to 4.5 mA cm^{-2}.

Supplementary References

S1. Li H, Hou Y, Wang F, Lohe MR, Zhuang X, Niu L, *et al.* Flexible all-solid-state supercapacitors with high volumetric capacitances boosted by solution processable MXene and electrochemically exfoliated graphene. *Adv. Energy Mater.* **7,** 1601847 (2017).

S2. Xu Y, Lin Z, Huang X, Liu Y, Huang Y, Duan X. Flexible solid-state supercapacitors based on three-dimensional graphene hydrogel films. *ACS Nano* **7,** 4042-4049 (2013).

S3. Xiao F, Yang S, Zhang Z, Liu H, Xiao J, Wan L, *et al.* Scalable synthesis of freestanding sandwich-structured graphene/polyaniline/graphene nanocomposite paper for flexible all-solid-state supercapacitor. *Sci. Rep.* **5,** 9359 (2015).

S4. Choi C, Lee JA, Choi AY, Kim YT, Lepró X, Lima MD, *et al.* Flexible supercapacitor made of carbon nanotube yarn with internal pores. *Adv. Mater.* **26,** 2059-2065 (2014).
