# Supplementary material for: Layer-by-layer self-assembly of pillared two-dimensional multilayers
Source: Nat Commun. 2019 Jun 11;10:2558. doi: 10.1038/s41467-019-10631-0 (PMC6560128; doi:10.1038/s41467-019-10631-0)
Supplement: Supplementary file 3 — Description of Additional Supplementary Files [file 41467_2019_10631_MOESM3_ESM.pdf]

Description of Additional Supplementary Files

File Name: Supplementary Movie 1
Description: (MXeneTAEA)20 on PET film under bending and twisting conditions connected into a circuit lighting up a LED.

File Name: Supplementary Movie 2
Description: (MXeneTAEA)30 on nonwoven under extreme knotted conditions connected into a circuit lighting up a LED.

File Name: Supplementary Movie 3
Description: The reversible compressing of (MXeneTAEA)10 on melamine resin.
